# Vicia ervilia lectin (VEA) has an antibiofilm effect on both Gram-positive and Gram-negative pathogenic bacteria

**DOI:** 10.1007/s00203-024-04100-6

**Published:** 2024-08-09

**Authors:** Beatrice Belfiori, Claudia Riccioni, Donatella Pietrella, Andrea Rubini, Maria Eugenia Caceres, Fulvio Pupilli, Michele Bellucci, Francesca De Marchis

**Affiliations:** 1 Institute of Biosciences and Bioresources (IBBR), National Research Council (CNR), 06128 Perugia, Italy; 2 Department of Medicine and Surgery, University of Perugia, Piazzale S. Gambuli 1, 06132 Perugia, Italy

**Keywords:** Lectin, *Vicia ervilia*, Biofilm, Antibiotic resistance, Antimicrobial, Lectin gene

## Abstract

Bacterial growing resistance to antibiotics poses a critical threat to global health. This study investigates, for the first time, the antibiofilm properties of *Vicia ervilia* agglutinin (VEA) from six different *V. ervilia* accessions against pathogenic bacteria, and the yeast *Candida albicans*. In the absence of antimicrobial properties, purified VEA significantly inhibited biofilm formation, both in Gram-positive and Gram-negative bacteria, but not in *C. albicans*. With an inhibitory concentration ranging from 100 to 500 µg/ml, the VEA antibiofilm activity was more relevant against the Gram-positive bacteria *Streptococcus aureus* and *Staphylococcus epidermidis*, whose biofilm was reduced up to 50% by VEA purified from accessions #5 and #36. VEA antibiofilm variability between accessions was observed, likely due to co-purified small molecules rather than differences in VEA protein sequences. In conclusion, VEA seed extracts from the accessions with the highest antibiofilm activity could represent a valid approach for the development of an effective antibiofilm agent.

## Introduction

The decreasing effectiveness of antibiotics and other antimicrobial agents has become a priority for global public health (World Health Organization 2020). It is estimated that by 2050 antibiotic-resistant infections could kill 10 million people worldwide per year (Kwon and Powderly 2021). Failures of antibiotic treatments are often related to bacterial biofilm formation, responsible for chronic infections, exacerbation as well as reinfection. The most common chronic infections are non-healing wounds generally associated with diabetics (Pouget et al. 2020), bed-bound and chair-bound patients (Dunyach-Remy et al. 2021), burn victims (Maslova et al. 2021), along with patients with traumatic and surgical wounds (Percival 2017). Common etiologic agents of wound infections are *Staphylococcus* and *Streptococcus* species, *Pseudomonas aeruginosa* and *Enterococcus species* (Scales and Huffnagle 2013). The aforementioned bacteria are all capable of forming microbial sessile communities living attached either to abiotic/biotic substratum or to each other, in a matrix composed of proteins, lipids and polysaccharides. In such communitarian structures, bacteria are more resistant to antimicrobial drugs and immune system responses in comparison to the planktonic form (Donlan and Costerton 2002). Standard protocols based on topical and systemic drug administration are often insufficient to remove established biofilms and the prevention of biofilm formation is the main goal in non-healing wound treatment. In the attempt to eradicate biofilms or prevent their formation, several strategies have been employed, each of which with advantages and disadvantages (Wu et al. 2015), and in this regard drug reshaping may represent a valid option. New therapeutic strategies contemplate the use of molecules that can support the activity of current-use antibiotics by interacting with bacteria, inhibiting the adhesion to epithelia and the consequent biofilm formation. For this reason, new anti-biofilm drugs have been investigated such as the quorum sensing inhibitor FS3 (Cirioni et al. 2013) or the inhibitors of diguanylate cyclase (Sambanthamoorthy et al. 2014). Significant antimicrobial activity has been demonstrated by a biosurfactant derived from *Streptomyces thinghirensis* showing growth inhibition of pathogenic bacteria including *E. coli* and *S. aureus* (Bellebcir et al. 2023). Nanoparticles has recently showed antibacterial properties with selenium nanoparticles significantly inhibiting *E. coli* and *S. aureus* from forming biofilm (Kandasamy et al. 2024). Nanotechnology has also been used for investigating the antifungal properties of Fe3O4@SiO2/Schiff‑base/Cu(II) magnetic nanoparticles, showing antimycotic activity against pathogenic *Candida* species (Azadi et al 2024). Plants natural compounds represent one of the most important sources of new drugs (Atanasov et al. 2015) and may constitute a promising alternative or adjuvant to antibiotics as antibiofilm agents. Extracts of the medicinal plant *Panax ginseng* seem to have negative effects on *P*. *aeruginosa* motility preventing biofilm formation (Wu et al. 2011). Being involved in immune defence against plant pathogens attacks, plant lectins emerge as candidates (Breitenbach Barroso Coelho et al. 2018) or adjuvants (Santos et al. 2021) in antimicrobial drugs production (Lannoo and Van Damme 2014).

Lectins are naturally occurring proteins/glycoproteins of non-immune origin that bind specific carbohydrates, present either in free form or as a part of glycoproteins and glycolipids, in a reversible and non-enzymatic manner. The term “lectin”, from Latin verb *legere* (which means “to select”), was proposed in 1954 by Boyd and Shapleigh after noticing the ability of these molecules to discriminate and agglutinate erythrocytes of different blood types (hence the other name “agglutinins”). Then lectins seem to agglutinate, in a specific manner depending on carbohydrates surface, other types of cells as well. By nature, carbohydrates belongs to a far more diverse group from proteins, being able to create very complicated branched structures, conferring to each cell, membrane or intracellular organelle its own uniqueness. These glucidic structures can be recognized by specific lectins in a way similar to the antibodies specificity in the immune system. Lectins have been largely employed in medicine to investigate the organization and function of complex carbohydrates in bio-recognition technology, to map changes in cell surface during physiological and pathological processes, to type blood cells and bacteria, to stimulate lymphocytes and to assess the immune state of patients (Bah et al. 2013).

Lectins occur in all living organisms, from bacteria to viruses, from fungi to animals, yet they are mainly extracted from plants. Since the discovery of the first plant lectin, ricin, more than 130 years ago, a crescent number of plant lectins have been characterized (Tsaneva and Van Damme 2020). In the plant kingdom, at least 12 lectin families are defined on the basis of their carbohydrate recognition domain (CRD) along with their sequence, structural homology and evolutionary relationships (De Coninck and Van Damme 2021). Among plants, the Leguminosae family, where lectins can reach a concentration as high as 10% of the total nitrogen in mature seeds (Etzler 1986), has been the most widely studied subject. Though exhibiting a considerable difference in carbohydrate binding specificity, largely due to variability around the conserved CRD, all legume lectins share a similar three-dimensional structure and high amino acid sequence correspondence (Lagarda-Diaz et al. 2017). Both anti-proliferative and antibiofilm activity on pathogenic bacteria have been reported for legume lectins. Phytohemagglutinin lectin (PHA), extracted from seeds of five *Phaseolus vulgaris* cultivars, have showed an antimicrobial activity against Gram-positive (*S. aureus* and S. *mutans*) and Gram-negative bacteria (*Klebsiella pneumonia* and *P. aeruginosa*), although at different degrees depending on the cultivar analysed (Hamed et al. 2017). Antimicrobial effects have also been reported for *Archidendron jiringa* (Charungchitrak et al. 2011)*, Indigofera heterantha* (Qadir et al. 2013), *Apuleia leiocarpa* (Carvalho et al. 2015) and *Cicer arietinum* (Gautam et al. 2018) lectins, all capable of inhibition against Gram-positive and Gram-negative bacteria. Lectins from legume seeds of different species (Teixeira et al. 2006) have been employed to prevent oral biofilm formation from pathogenic bacteria responsible for caries and periodontitis, as also shown in *Bauhinia variegata* (Klafke et al. 2013), in some *Canavalia* species (Cavalcante et al. 2011), and other legumes like *Phaseolus vulgaris* and *Pisum sativum* (Islam et al. 2009).

Among Leguminosae, lectins of the Fabeae tribe (formerly referred as Vicieae) have been identified to be strongly reactive towards the components of the bacterial cell wall peptidoglycan (PGN) through the same interactions employed to bind monosaccharides (glucose and/or mannose) and their derivatives (Ayouba et al. 1994). Mannose-binding lectins (MBLs) are considered as potent anti-pathogenic proteins, constituting protective tools in plants (Barre et al. 2001) and animals to fight microbes (Dos Santos Silva et al. 2019). *Vicia ervilia* (L.) Willd., known as bitter vetch, is an annual legume species belonging to the *Vicia* genus of the Fabeae tribe. The MBL *Vicia ervilia* agglutinin (VEA) was first extracted and characterised from bitter vetch back in Fornstedt and Porath (1975). VEA is produced by the pharmaceutical industry and largely employed as biospecific adsorbent for virus purification and in membrane protein studies, but its effect on pathogenic bacteria has never been investigated. VEA ability to bind to mannose and glucose monosaccharides in the cell wall leads to its potential property of preventing microbial biofilm formation, a critical factor in bacterial virulence and persistence. Hypothesizing that VEA may be used as an antibiofilm agent, we have, in this study, investigated VEA capacity to prevent biofilm formation responsible for human non-healing wounds.

We extracted VEA from six different *V. ervilia* accessions of the Mediterranean basin, all chosen basing on their previous genetic characterization (Russi et al. 2019), to evaluate if the intraspecies genetic diversity can affect VEA antibiofilm capacity. The inhibitory effect on the initial attachment to solid surfaces of Gram-positive and Gram-negative bacteria (*S. aureus*, *S. epidermidis* and *P. aeruginosa*), and the polymorphic fungus *Candida albicans*, involved in non-healing wounds was taken into exam. VEA effects were analysed on both Gram-positive and Gram-negative bacteria because of their different cell wall structures and biofilm formation mechanisms. The results demonstrated the efficiency of VEA against aforementioned different bacterial types, highlighting its potential as a broad-spectrum antibiofilm agent or adjuvant to complement existing treatments.

## Materials and methods

Unless otherwise noted, all chemicals were of analytical grade and were obtained from Merck KGaA, Darmstadt, Germany.

### Plant materials

Seeds from six bitter vetch accessions were employed for lectin extraction. Accessions #5 and #12 were provided by the Aegean Agricultural Research Institute, Turkey. Accession #21 was an Italian landrace maintained and multiplied by the Gerace & Giunti Farm in Tuscany region. Accession #23 consisted of natural populations collected in Central Italy and conserved at the Germplasm Bank of Department of Agricultural, Food and Environmental Sciences (DSA3), University of Perugia, Italy. Accession #36, a Cyprus local landrace, was supplied by the Agricultural Research Institute, Lefkosia, Cyprus. Accession #46, a landrace collected in Spain, was provided by the Centro de Recursos Fitogeneticos, INIA, Madrid.

### Extraction of VEA from V. *ervilia* seeds

Powders obtained by grounding ten grams of *V. ervilia* seeds were extracted with PBS (1:8, w/v) for 48 h at 4 °C with continuous stirring. After filtration through Miracloth (Merck KGaA, Darmstadt, Germany), the filtrates were centrifuged at 7400 × g for 30 min. In order to eliminate seed storage proteins, which precipitate at low pH values, the clarified crude extracts (CEs) were adjusted to pH 4.5 by slowly adding acetic acid 1 M. Samples were maintained under constant stirring at 4 °C for 30 min before centrifugation at 7400 × g for 30 min, at 4 °C. After centrifugation, pellets were discarded and the resulting supernatants were readjusted to pH 7.5 with NaOH 1 M before being fractionally precipitated with ammonium sulphate at 30%, 70% and 90% saturation, respectively. After each ammonium sulphate precipitation step, pellets, resulting from centrifugation at 7400 × g for 30 min at 4 °C, were dissolved in a minimal volume of PBS, and dialyzed for 36 h against PBS by replacing buffer at 12 h intervals. For all the dialysed samples, as well as for all the CEs, protein concentration was evaluated by Bradford’s assay (Bradford 1976) before performing the haemagglutination assay.

### Purification of VEA by affinity chromatography

VEA was further purified by affinity chromatography. The dialysed 30–70% ammonium sulphate fractions were loaded on a Sephadex G-100 column (1.6 × 15 cm) equilibrated with PBS. Once loaded, the unbound material was washed with the same buffer at a constant flow rate until the 280 nm absorbance of the collected fractions reached the zero value. The retained lectin was eluted with 0.1 M D-glucose in PBS until the effluent absorbance at 280 nm was stabilised to zero. Fractions with the highest absorbance were pooled and dialyzed for 36 h against deionized water by replacing water at 12 h intervals. The dialysates were lyophilised, resuspended in PBS and analysed by both hemagglutination assay and 15% acrylammide SDS-PAGE, followed by Coomassie staining (Brunelle and Green 2014), to verify lectin presence and purity, respectively. Purified lectins were tested for antibacterial and biofilm growth inhibitory effects.

### Hemagglutinating activity assay

Hemagglutination assays were carried out using normal human ABO erythrocytes in V-bottom 96-well microtiter plates as previously described with some modifications (Liu et al. 2008). All dilutions were performed in Alsever’s solution (citric acid 0.055 g, sodium citrate 0.8 g, D-glucose 2.05 g, sodium chloride 0.42 g in 100 ml of distilled water). Twenty µl of each sample (CE, precipitated 30–70% fraction or the purified lectin) were placed in the first well and serially diluted (twofold dilution). Twenty µl of 2% erythrocyte suspension were then added. The plate was incubated at 37 °C for 30 min and then for 1–2 h at 4 °C. The hemagglutination titer was scored visually. The reciprocal of the highest sample dilution showing complete agglutination was taken as hemagglutination titer.

### Microorganisms

The microbial strains used in this study consisted of two Gram-positive bacteria *S. aureus* (ATCC 25923) and *S. epidermidis* (ATCC 35984), the Gram-negative* P. aeruginosa* (ATCC PAO-1) and the yeast *C. albicans* (strain SC5314 / ATCC MYA-2876). Bacterial cultures were maintained in Mueller Hinton Agar (MHA). *C. albicans* was maintained in Sabouraud agar (SAB). The day before the test, one colony was inoculated in 7 ml of Mueller Hinton broth (MHB) or SAB broth and incubated for 24 h at 37 °C. The strains used came from the Department's microbial strains bank (Department of Medicine and Surgery, University of Perugia).

### Minimal inhibitory concentration (MIC) assay

The Minimal Inhibitory Concentration (MIC) was determined by micro broth dilution method according to the Clinical and Laboratory Standards Institute/National Committee for Clinical Laboratory Standards (CLSI/NCCLS) Approved Standard M100-S21, 2007 (Clinical and Laboratory Standards Institute). Determination of MIC against microbial strains was determined by broth microdilution assay using two-fold serial dilutions in Muller Hinton Broth for bacteria and RPMI 1640/MOPS for the yeast *C. albicans*. The test was performed in 96-well U-bottom microdilution plates. Microbial inocula were prepared by subculturing bacteria into Muller Hinton Broth (MHB) and *Candida* cells in Sabouraud Broth at 37 °C overnight, and then diluted to approximately 10^5^ – 10^6^ CFU/ml in MHB or RPMI/MOPS. One hundred μl of crude extract or the purified lectin were diluted 1:2 in appropriate medium (1000, 500, 250, 125, 62.5, 31.5, 15.6, 7.8, 3.9, 1.8, 0.9, 0.45, 022 mg/L) and placed in a 96-well tissue culture plate. One hundred μl aliquots of test microorganisms were added to each well. Microplates were then incubated at 37 °C for 24 h. MIC value was defined as the lowest concentration of compound inhibiting microbial growth. As positive growth control, wells inoculated with microorganisms in the absence of the tested compound were carried out. The positive control for Gram-positive and Gram-negative was gentamicin, and fluconazole for *C. albicans*. Each experiment was repeated at least three times.

### Biofilm formation determination using crystal violet staining

The in vitro static biofilm assay was performed using a 96-well flat bottom microtiter plate as previously described with some modifications (Bakke R. 2001). To grow biofilms, overnight cultures of bacteria in MHB or yeast in SAB broth were diluted 1:100 into fifteen mL of MHB or SAB broth supplemented with 2% sucrose, in presence or in absence of crude extract or the purified lectin at the concentrations of 500 and 100 µg/ml. Cultures were incubated at 37 °C for 24 h under static conditions. After incubation, the biofilm developed in each well was washed twice with 200 μl of distilled water and then dried for 45 min. One hundred μl of 0.4% crystal violet were added to each well for 30–45 min. The wells were then washed four times with distilled water and immediately discolored with 200 μl of 95% ethanol. After 45 min, 100 μl of discolored solution were transferred to a well of a new plate and the crystal violet measured at 570 nm in a microplate reader (Infinite M200 pro, TECAN, Männedorf, Switzerland). The amount of biofilm mass was measured comparing the absorbance values of the crude extract-treated, or the purified lectin-treated, wells versus untreated control wells. Biofilm formation bioassays were performed in triplicate in at least three individual experiments for each concentration. The positive control for Gram-positive and Gram-negative bacteria was gentamicin and fluconazole for *C. albicans*. Lectin activity was compared with phytohaemagglutinin (PHA) of *Phaseolus vulgaris* at the concentration of 50 and 100 µg/ml.

### VEA gene cloning and sequencing

Plant genomic DNA was extracted from 0.1 g of young leaves of accession #12 using the HiPurA Plant Genomic DNA Miniprep Purification kit (HiMedia, Mumbai, Maharashtra, India). After a database search for sequence homology between lectin genes of species belonging to the *Fabeae* tribe, a set of primers in the 5' UTR (5’-catgcatgcatgcaattattaccaa-3’) and 3' UTR (degenerate primer, 5’-grygrgaagcyraaaactawgca-3’) regions was designed and used for PCR amplification. A single band of approximately 850 bp was obtained, cloned into pGEM-T Easy plasmid (Promega, Madison, Wisconsin, USA) and sequenced to define the VEA gene sequence from the ATG to the stop codon.

Based on this sequence, two further primers were designed at 5', Vea_fw (5’-atggcttccattcaaacccaaatgatttc-3’), and 3', VEA_rw (5’-ctaagcagatgtagcttggttataacttg-3’), of the *V. ervilia* lectin gene. These primers were used to amplify the VEA gene from all six accessions. A single band of 828 bp was obtained in all samples which, after purification using NucleoSpin Gel and PCR Clean-up (Macherey–Nagel, Dueren, Germany), was analysed by direct sequencing to check for any differences between them. The VEA lectin gene sequence was deposited in the GenBank with the following accession number: PP845299.

## Results and discussion

### VEA purification from seeds of six V. *ervilia* accessions and hemagglutination activity

Since the discovery of penicillin in 1928, antibiotics have revolutionized modern medicine and saved millions of lives, but the large use of antibiotics in the world, recently exacerbated by the massive treatments in SARS-CoV-2 infected patients, have speeded up the antimicrobial resistance ongoing threat (Kariyawasam et al. 2022). The formation of bacterial biofilms is often responsible for the absent antibiotic activity of medical drugs, so plant lectins offer a potential alternative treatment strategy. Lectins antibiofilm activity from four leguminous plants and two red algae has been reported by Vasconcelos and colleagues (2014) towards clinically relevant microorganisms, and the bacterial antibiofilm properties of *Canavalia ensiformis* (Jin et al. 2019) and *Musa acuminata* (Ahmed et al. 2023) lectin have also been described.

To evaluate VEA action on bacterial biofilms responsible of non-healing wounds, seeds of six *V. ervilia* accessions from the Mediterranean area have been utilised in this study as lectin sources, because different cultivars/accessions of the same legume species can, as previously reported, produce seed lectins with variable activities. PHA lectins extracted from *P. vulgaris* seeds have shown distinct antimicrobial capacities depending on their cultivar origin (Hamed et al. 2017). Lectins isolated from the seeds of three *P. vulgaris* cultivars also have different anticancer properties, ranging from significant antiproliferative activity towards hepatoma HepG2 cells (Fang et al. 2010) to mild inhibition of HepG2 cell growth (Chan et al. 2012) to no anticancer activity (Sharma et al. 2010).

**Fig. 1:**
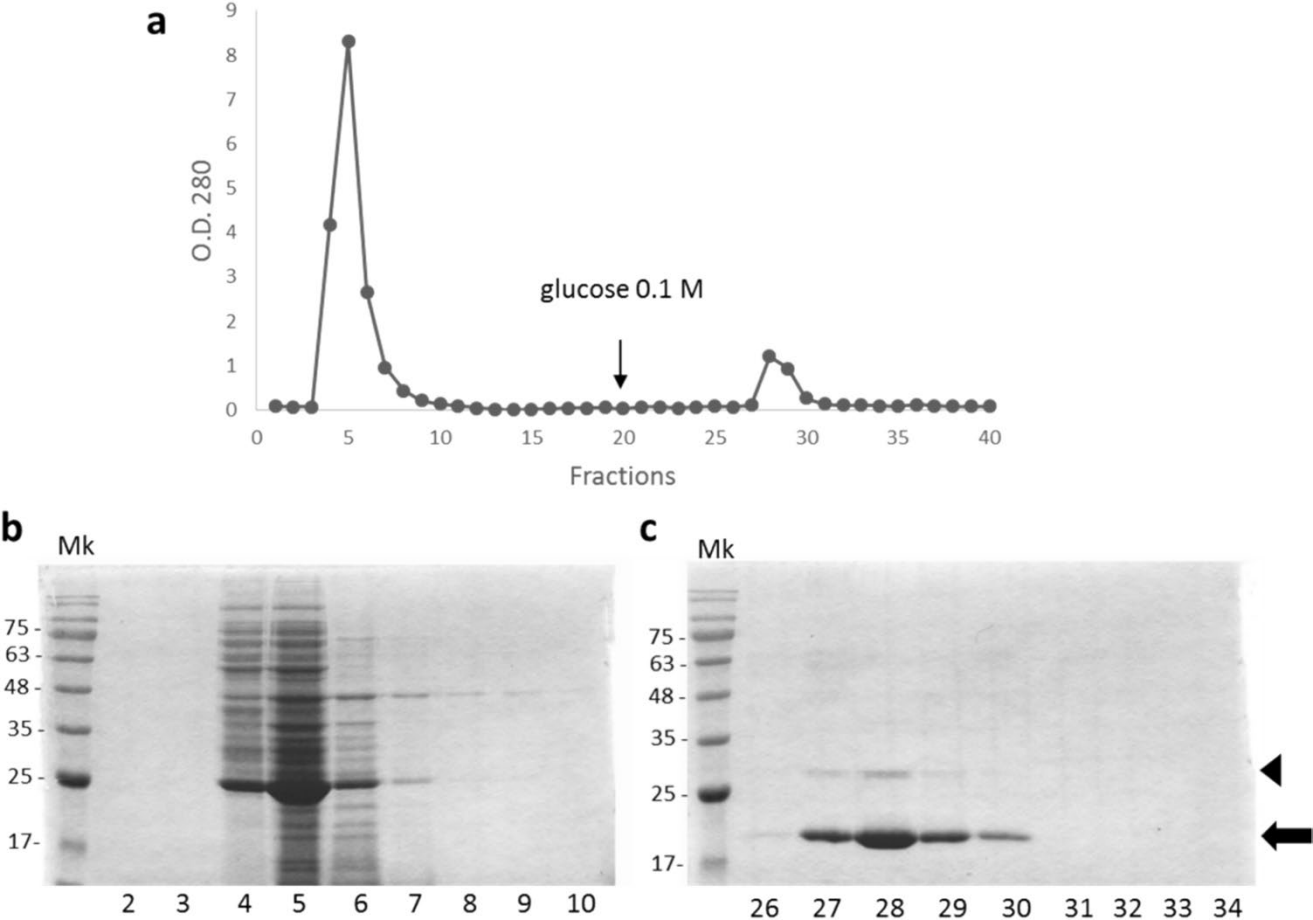
Chromatogram of VEA purification trough affinity chromatography. **a** Example of affinity chromatography elution profile at 280 nm for one of the six *V. ervilia* accessions utilized in this work. The first peak (fraction 3–10) corresponds to unbound proteins eluted from the column with PBS buffer. The second peak (fractions 27–31) results from protein elution after the addiction of PBS buffer with 0.1 M glucose to the column. **b-c** SDS-PAGE followed by Coomassie staining of the first (**b**) and the second (**c**) affinity chromatography peak. Numbers of the affinity chromatography fractions analyzed by SDS-PAGE are reported below gels. Twenty µl of each elution fraction were loaded on a 15% polyacrylamide denaturing gel. The black arrow marks the 21-kDa beta-chain subunit of the purified VEA; the arrowhead indicates a protein of around 30 kDa that likely represents uncleaved lectin precursor. Numbers at left indicate molecular mass markers (Mk) expressed in kDa

**Fig. 2:**
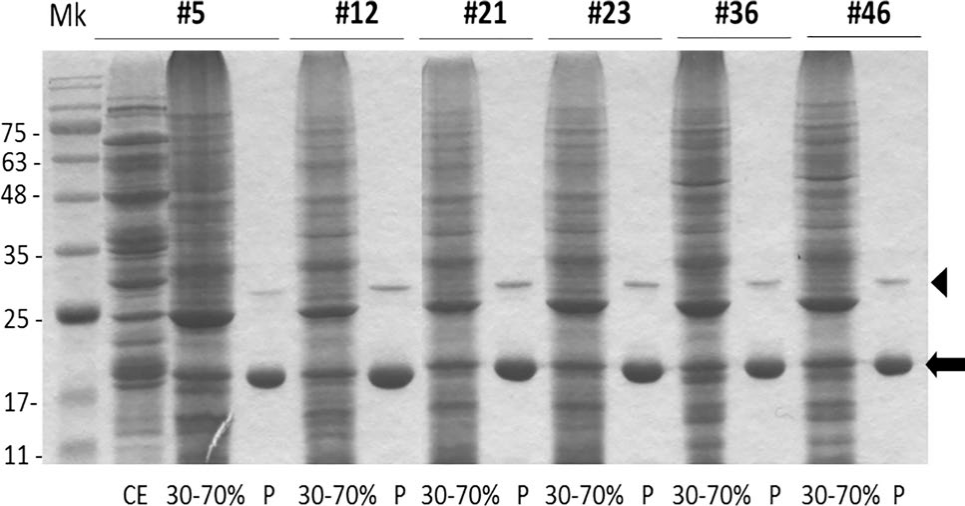
SDS-PAGE analysis to confirm VEA purification for all the six *V. ervilia* accessions (#5, #12, #21, #23, #36, #46). Twenty µg of proteins from crude extract (CE) (in figure reported only for the #5 accession, as an example), or from 30–70% ammonium sulphate precipitation samples, or two µg of proteins purified by affinity chromatography (P), were loaded on a polyacrylamide gel and elettrophoretically separated before Coomassie staining. Black arrow: 21-kDa subunit of the purified VEA. The arrowhead indicates a protein of around 30 kDa that likely represents uncleaved lectin precursor. Numbers at left indicate molecular mass markers (Mk) expressed in kDa

Acid acetic and ammonium sulphate precipitation represented the first passage of bitter vetch lectin purification, as described in Materials and Methods. Based on haemagglutination results, the 30–70% precipitation fraction was individuated as the one containing VEA (data not shown) and was loaded on a Sephadex G-100 column for further purification (Fig. 1a-c). Two peaks were visible in the affinity chromatography elution profile (Fig. 1a). The first one resulted from elution of unbound proteins to the column, which were visualised in Fig. 1b as multiple bands of different molecular weights after an SDS-PAGE and Coomassie staining. The second peak contained the 21-kDa VEA that was eluted after the addiction of PBS buffer with 0.1 M glucose to the column (Fig. 1c). *V. ervilia* lectin was described as a heterotetramer two-chain protein whose subunits, identical pairwise, had a molecular weight of 21 and 4.7 kDa, respectively (Fornstedt and Porath 1975). In Fig. 1c, the 21-kDa larger beta-chain subunit was detectable in a polyacrilammyde gel after electrophoretic protein separation, whereas the 4.7 kDa alpha-chain subunit failed to be detectable, not even with a shorter gel run performed (data not shown). Another protein of around 30 kDa was also evident in Fig. 1c, which could be a contaminant protein or, most likely, representing the residual amounts of still uncleaved lectin precursor (its proteolytic maturation originates the alpha-and beta-chains), as reported in *Lens culinaris* (see Fig. 1B of Galasso et al. 2004). The purification protocol was repeated for all the six bitter vetch accessions reaching a significant level of purification (Fig. 2).

To confirm the presence of VEA lectin in the samples, we monitored, during the entire process of protein purification, the extracts capacity to retain the hemagglutinating property. The hemagglutination activity of crude extracts, 30–70% ammonium sulphate precipitated and purified lectins (P) was determined by a twofold serial dilution using human erythrocytes of the ABO system. Common bean (*Phaseolus vulgaris*) phytohaemagglutinin (PHA), known to have a stable hemagglutination activity (Peddio et al. 2023), was used as positive control. For all the six *V. ervilia* accessions tested, purified lectins showed a high hemagglutination activity (Table 1) with respect to the PHA control, demonstrating that VEA could agglutinate human erythrocytes.

**Table 1:** Determination of hemagglutination titer of crude extracts, 30–7% precipitated and purified lectins from different accessions

Hemagglutination titer			
Accession number	CE	30/70%	Purified lectin (P)
#5	32	64	2048
#12	32	256	2048
#21	32	256	2048
#23	32	128	2048
#36	32	256	2048
#46	32	128	4056
PHA	–	–	64

Twenty μl of crude extract (CE), precipitated 30–70% or the pure lectin (P, 10mg/ml) were twofold serially diluted and 20 μl of 2% human erythrocyte suspension were added. The plate was incubated at 37℃ for 30 min and then for 1–2 h at 4℃. PHA (100 μg/ml) was added to the experiment as a positive control. Data represent the mean of three independent evaluations

Based on hemagglutination data, we evaluated the VEA purification degree starting from the crude extract (CE), obtained from 10 g of seeds, to the affinity chromatography P fractions. Table 2 reported the results for three representative *V. ervilia* accessions, one for each geographic origin. The values were similar for these accessions: the purification grade of the P fractions ranged from 142 to 261 folds, with an increase of specific activity from about 2 units/mg in the crude extract to more than 300 units/mg after the final affinity chromatography, indicating that our P fractions were enriched in functional VEA lectin.

**Table 2:** Purification of VEA from 10gr of seeds of three reperesentative *V. ervilia* accessions

	Lectin fraction	ml	[Protein] mg/ml	Total Protein (mg)	Total lectins (HU) (titrer^a^X ml)	Specific activity^b^ (HU/mg)	Purification degree
#12 (Turkey)	CE	60	16.4	984	1920	1,95	1
	30%-70%	6	10	60	1536	25,6	13
	P	0,6	6.7	4	1229	307	157
#21 (Italy)	CE	63	17	1071	2016	1,88	1
	30%-70%	6	12	72	1536	21,3	11
	P	0,6	4.1	2.5	1229	491,6	261
#36 (Ciprus)	CE	61	14	835	1952	2,33	1
	30%-70%	6	12	72	1536	21,3	9
	P	0,6	6.2	3,7	1229	332	142

^a^ Hemagglutination titer is the reciprocal of the greatest dilution of the solution that cause complete agglutination of the red blood cells. ^b^ Specific activity is defined as the hemagglutination unit (HU) divided by thr total protein content of the sample used for the assay

CE, crude extract; 30–70%, ammonium sulphate precipitation fraction; P, affinity chromatography purified protein

### Effect of VEA on the bacterial growth

In order to explore the biological activity of the bitter vetch lectin on pathogenic microorganisms, we tested the effect of VEA, from all the six accessions, for its antibiotic and antibiofilm properties on the Gram-negative *P. aeruginosa*, and two Gram-positive *S. aureus* and *S. epidermidis* bacteria that are of high medical relevance and cause serious infections in humans. The yeast *C. albicans* was also included in the species tested because it leads to severe diseases and systemic infections in immunocompromised patients (Pappas et al. 2018).

The six *V. ervilia* accessions used in this study belonged to traditional varieties (landraces), genetically heterogeneous and cultivated in specific geographical areas of Italy, Turkey, Cyprus and Spain. Despite these different geographical origins, the purified VEA proteins from these landraces shared similar characteristics, all showing both high haemagglutination capacity and lack of antibiotic activity. As reported for other lectins (Procopio et al. 2017), VEA did not act as an antibiotic because none of the six CEs or P fractions showed any antimicrobial activity, and cell growth occurred even at the highest concentration of 1000 µg/ml for all samples (data not shown). Nevertheless, agents without any effect on bacterial survival and with specific impact on biofilm growth were considered more relevant, as the emergence of microbial resistance to these molecules would be minimised (Rabin et al. 2015).

**Fig. 3:**
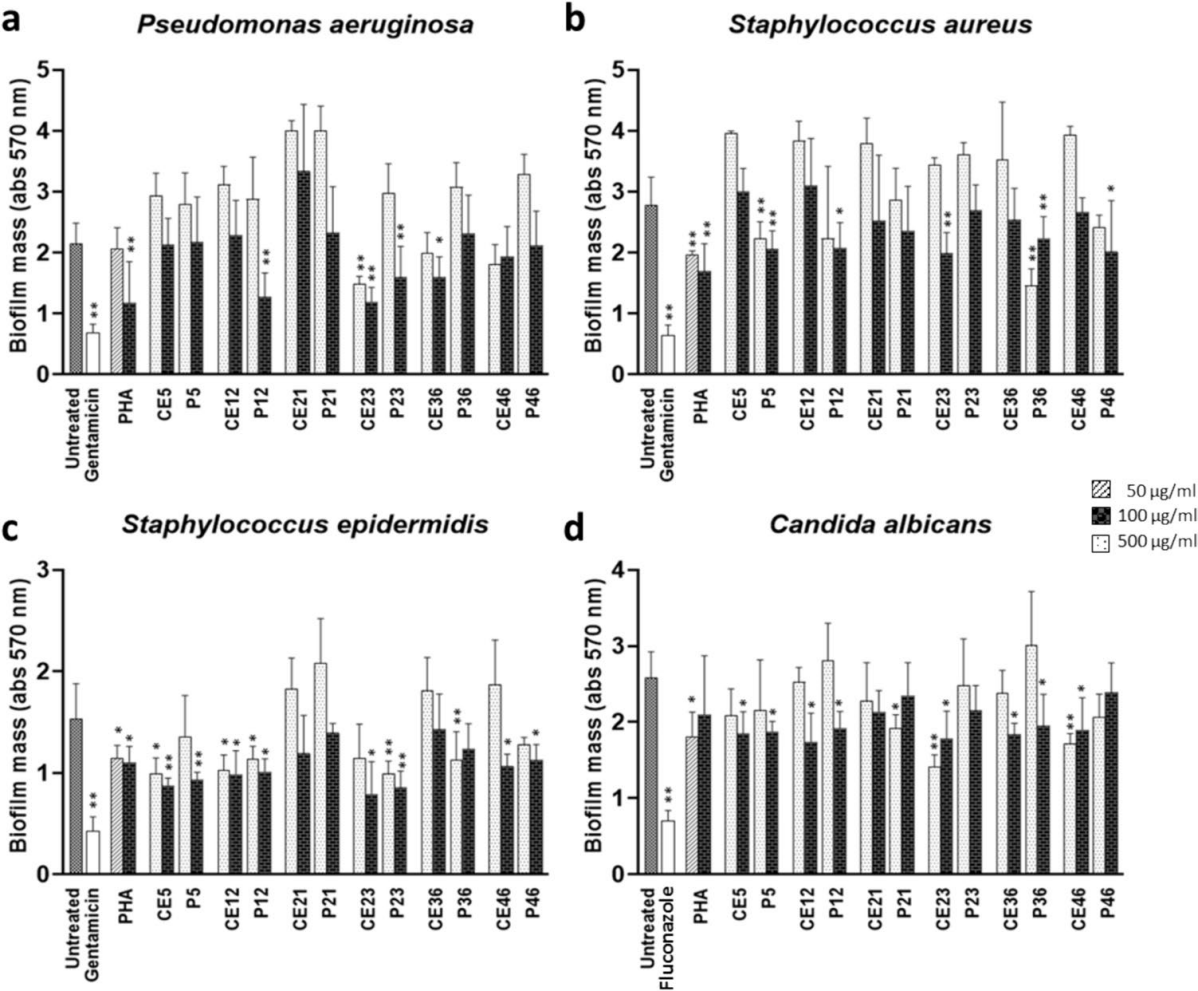
The effect of VEA lectins on biofilm formation. *P. aeruginosa* (**a**), *S. aureus* (**b**), *S. epidermidis* (**c**), *C. albicans* (**d**) were inoculated into a 96-well plate containing crude extract (CE) and purified VEA lectin (P) at 100 or 500 µg/ml and incubated for 24 h. Biofilm biomass was quantified by crystal violet assay (absorbance 570 nm). As positive control, gentamicin, and fluconazole for the yeast, were used. Antibiofilm lectin activity was even determined with PHA at the concentration of 50 and 100 µg/ml. Data represent the mean ± SD of three independent experiments performed in quadrupled. *P < 0.05, **P < 0.01 (treated versus untreated microorganisms)

Several legume lectins, though not always able to reduce bacterial planktonic growth, can still influence Gram-negative and Gram-positive biofilm formation, causing a decrease in their biomass, as demonstrated in *Canavalia ensiformis* (Jin et al. 2019) and *Musa acuminata* (Ahmed et al. 2023). The induction of large microbial aggregates, associated with lectin action, actually causes a decrease in the number of bacteria adhering to surfaces, probably explaining why lectins inhibit biofilm formation but do not reduce bacterial growth. CEs and P fractions of the accessions #5, #12, #21, #23, #36 and #46 were tested for their antibiofilm activity against the aforementioned microorganisms at the concentrations of 100 and 500 µg/ml. As a positive control, gentamicin for bacteria and fluconazole for the yeast were used. Antibiofilm lectin activity was even determined with PHA at the concentration of 50 and 100 µg/ml. With respect to biofilm formation in *P. aeruginosa* (Fig. 3a), only the purified lectins from accession #23 and #12, at the concentration of 100 µg/ml, showed a significant inhibitory activity (P < 0.01) in comparison to the untreated control. At the concentration of 500 µg/ml, this effect disappeared, most likely because at the highest concentration proteins were incorporated into the forming biofilm of *P. aeruginosa*, thus increasing the mass of the biofilm itself. This phenomenon was even more evident when the concentration of purified lectin was increased to 1000 µg/ml, confirming our hypothesis and leading us to limit our analysis to 100–500 µg/ml (data not shown). Both CEs of accessions #23 and #36 were able to reduce the Gram-negative- bacterial biofilm, although to a different extent. In accession #36 this ability was lost with subsequent purification, suggesting that, at least for this sample, the activity observed in the CE was plausibly due to other molecules present in the mixture. For this reason, results reported hereafter will exclude samples with antibiofilm activity detected only in the CE samples but not in the P fraction.

The VEA antibiofilm activity against the Gram-positive bacteria *S. aureus* and *S. epidermidis* is shown in Fig. 3b and c. Purified lectins P5, P12, P36 and P46 were all able to reduce biofilm formation in both bacterial species, although the reduction was much more relevant for accessions #5 and #36 with biofilm mass reduced up to 50% in *S. epidermidis* and *S. aureus*. For the sample P36, contrary to what observed so far for other accessions, the degree of biofilm inhibition increased with the rise in purified lectin concentration (from 100 µg/ml to 500 µg/ml), suggesting that, at least for Gram-positive microorganisms, this protein was not incorporated into the growing bacterial biofilm. The purified lectin P23, able to reduce *P. aeruginosa* but without effect on *S. aureus* biofilm growth, actually showed good antibiofilm activity also on *S. epidermidis* reaching 50% of biomass inhibition. Regarding the activity of VEA on Gram-negative and Gram-positive bacteria, number #21 was the only accession that never showed antibiofilm activity, neither in CE nor in P samples. VEA action on the yeast *C. albicans* was present in all the purified samples, but, except for accession #23 and #46, it was mild compared to bacteria (Fig. 3d).

To summarize the results, we demonstrated that VEA did not exhibit antibiotic properties and its antibiofilm activity became evident only versus bacteria, especially the Gram-positive species. Both accessions #5 and #36 had a relevant antibiofilm effect against Gram-positive bacteria regardless of the species (*S. epidermidis* and *S. aureus*), conversely only two accessions, #12 and #23, significantly succeeded in inhibiting the Gram-negative *P. aeruginosa* biofilm. VEA antibiofilm activity against the yeast *C. albicans* was very low or absent in all accessions, as expected, because *C. albicans* adhesion is known to be a multifunctional system that allows the yeast to effectively adhere to many cell and tissue types, using hydrophobic or electrostatic forces to form biofilms (Chaffin 2002). We observed that the ability to inhibit biofilm growth varied according to the combination of accessions and microorganisms. The Turkish accession #12 was the only one that had always inhibited the biofilm mass of all the tested microorganisms, while the Italian accession #23, the Turkish accession #5, and the Cypriot accession #36 significantly inhibited the biofilm mass of two out of three bacterial species, in different VEA/species combination (e.g. P5 was able to reduce biofilm formation of both *S. epidermidis* and *S. aureus*, whereas P23 had the same effect in *P. aeruginosa* and *S. epidermidis*). The effective concentration of purified VEA protein to act as an antibiofilm agent was 100 µg/ml for four accessions (#5, #12, #23, #46) and 500 µg/ml for accession #36, in line with the lectin concentration of 100 µg/ml that inhibited the adherence of streptococci species to acquired pellicle in vitro (Teixeira et al. 2006), or with the 100–500 µg/ml concentration range of concanavalin A (ConA) used to inhibit enterohemorrhagic *E. coli* (EHEC) biofilms (Jin et al. 2019). VEA of accession #21 failed to inhibit bacterial biofilm formation at these concentrations.

### VEA sequence analysis

**Fig. 4:**
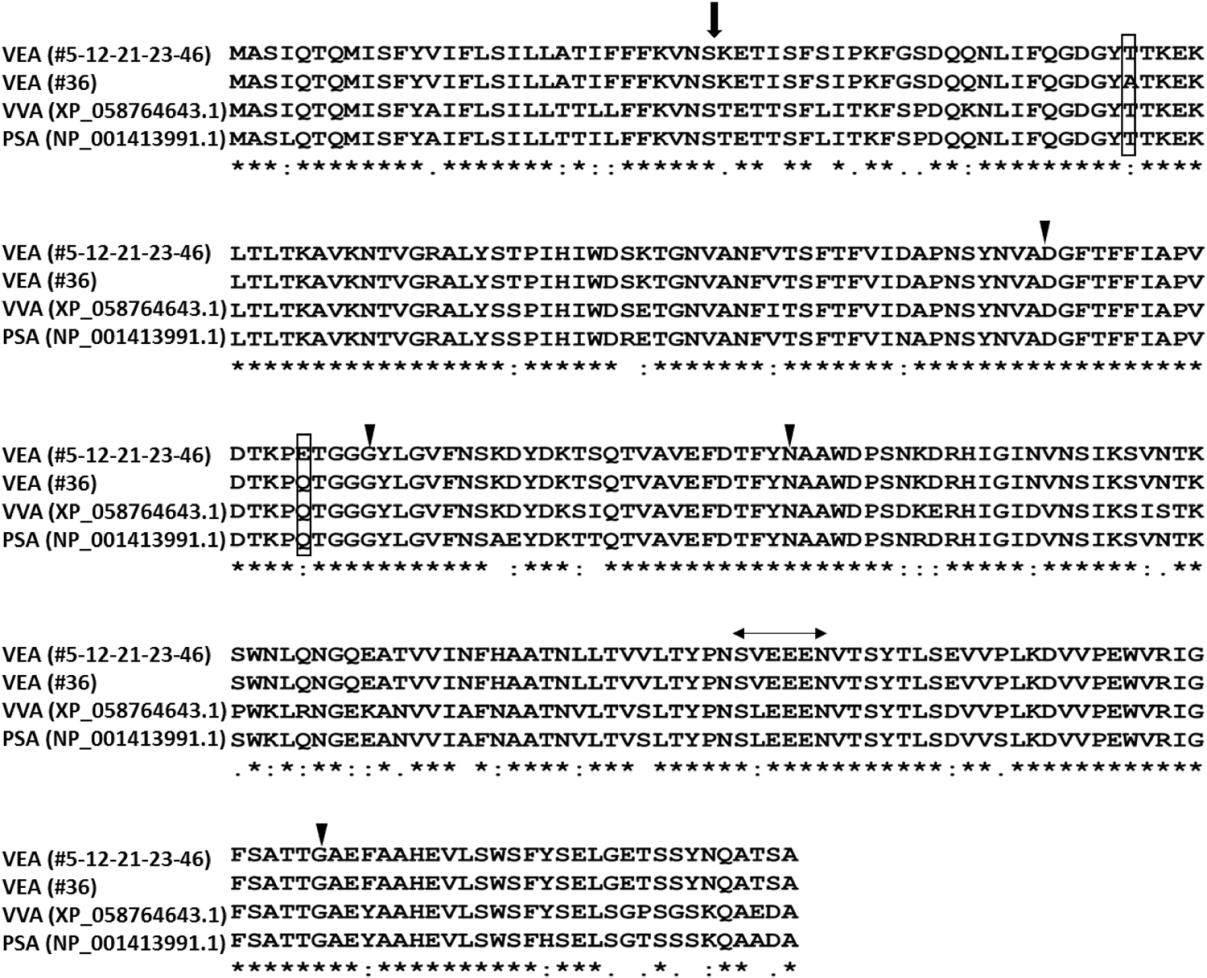
Protein alignment of lectin amino acid sequences. Lectin proteins from two species of the Fabeae tribe, VVA from *Vicia villosa* and PSA from *Pisum sativum*, are shown for comparison. The arrow indicates the internal cleavage site for removal of the signal peptide. In the rectangle, the A (Ala) and Q (Glu) substitutions in accession #36 are reported with respect to the other *V. ervilia* accessions (#5–12-21–23-46) that have an identical amino acid sequence. Harrowheads indicate conserved amino acid residues for carbohydrate binding. The SL/VEEN stretch, marked by two arrows, represents the six amino acid peptide between the mature protein alfa and beta chains. “*”: amino acidic residues are identical in all sequences in the alignment; “:” conserved substitutions, i.e. the amino acid is replaced by one having similar characteristics;”.” semi-conserved substitutions, i.e., amino acids having similar shape

To determine whether the differences in antibiofilm capacity detected in the six accessions were due to diversity in the amino acid sequence of VEA, we decided to PCR-amplify and sequence the *V. ervilia* lectin genes of the six landraces. As the *V. ervilia* lectin nucleotide sequence was unknown, the gene was first cloned with degenerate primers from genomic DNA of accession #12 as described in Materials and Methods. Based on this sequence, new specific primers were designed, and PCR amplification gave a single band of 828 bp in all six samples, corresponding to a protein of 275 amino acids. As reported for other legume lectins (Van Damme et al. 1998), the gene contains no intron and direct sequencing of the PCR amplicons reveal only one lectin DNA sequence for each accession, indicating the presence of a unique VEA gene. No lectin-related gene or pseudogene were amplified, but their presence in the genome could not be excluded. The multiple alignment of the VEA protein sequences with two lectin proteins representative of species belonging to the Fabeae tribe (Fig. 4), showed that residues essential for carbohydrate binding were conserved, together with the six amino acid stretch between the beta and alpha chains, subsequently removed in the mature lectin protein (Lioi et al. 2006). Only accession #36 had two different amino acids compared to all other bitter vetch accessions: A (Ala) to T (Thr) and Q (Glu) to E (Gln) at positions 56 and 125, respectively (Fig. 4). Q125E is close to a carbohydrate-binding site conserved in the Fabae tribe. In all legume lectins, about 20% of the amino acid residues are identical and a further 20% are similar; the conserved residues include many of those required for interaction with the sugar. While the invariant amino acids provide a skeleton for sugar binding, the specificity of individual lectins is most likely due to sequence variability in the proximal regions of the carbohydrate-binding site (Ambrosi et al. 2005). Accession #36 did not behave differently from the other samples in terms of biofilm inhibition capacity. The only difference lied in its visible dose-dependent effect: the concentration increment of purified VEA from accession #36 enlarged the magnitude of the antibiofilm effect on both Staphylococcus species, but this result was masked for the lectins purified from the other accessions, because they were likely incorporated into the growing biofilm.

The VEA sequences of the accessions studied here turned out to be indentical at both nucleotidic and amino acidic level, except for accession #36 with two different amino acids out of 275, indicating that the significant inter-sample variability observed in the biofilm growth inhibition was unlikely due to a corresponding variability in the VEA amino acid sequences. Other factors must be taken into account to explain why VEA ability to inhibit bacterial biofilms can vary greatly between accessions. According to Jin and colleagues (2019), their laboratory purified ConA from *C. ensiformis* (jack bean) reduced EHEC biofilm to a greater extent than commercial ConA. This effect was not present against *Listeria monocytogenes* biofilm, where both purified and commercial ConA showed a similar antibiofilm activity. The authors demonstrated that their laboratory ConA sample contained small molecules (< 10 kDa) of non-proteic origin, not present in commercial ConA, which specifically reduced EHEC biofilms and speculated that these small molecules could be co-purified carbohydrates from the jack bean, already bound to ConA. Polysaccharides have been shown to antagonistically reduce pathogen adhesion to the host cell surface (Wittschier et al. 2007). The presence of different small molecules in the six purified bitter vetch lectin extracts may be the reason for the observed variability in their antibiofilm activity, which is not explained by a diversity in their amino acid sequence and carbohydrate-binding sites. We think that in order to exploit the antibiofilm activity of a lectin, at least for legumes, preliminary testing on the ability of different accessions/varieties of the same species is a main factor to be considered.

## Conclusions

Several lectins from plant species, many of which belong to the Leguminosae family as the tropical shrubs *Calliandra surinamensis* (Procopio et al. 2017) and *Canavalia brasiliensis* (Cavalcante et al. 2011), have been shown to possess anti-biofilm activity (Ahmed el al. 2023). Although *V. ervilia* agglutinin has long been used in medical diagnostics, especially in conjugated form, as a biospecific adsorbent for analysis and membrane protein studies, this is the first demonstration of its antibiofilm activity against both Gram-positive and Gram-negative pathogenic bacteria. Effective VEA concentration ranged from 100 µg/ml to 500 µg/ml, in line with previous reports from other studies on Gram-positive (Teixeira et al. 2006) and Gram-negative microorganisms (Jin et al. 2019). The observed VEA antibiofilm activity was more relevant against the Gram-positive bacteria *S. aureus and S. epidermidis*, in fact, purified lectins P5 and P36 succeeded in reducing biofilm mass up to 50% in these pathogenic bacteria. The major cell wall component of Gram-positive bacteria is PGN, and lectins of leguminous plants are strongly reactive towards PGN (Ayouba et al. 1994), as well as human MBL binds significantly to PGN via its CRDs (Nadesalingam et al. 2005). VEA is a leguminous MBL, thus we speculate that VEA may preferentially interact with the PGN present in the Gram-positive bacteria cell wall.

To achieve a broader spectrum antibiofilm response, VEA should be extracted from a mixture of seeds belonging to accessions #5, #23 and #36. The addition of the crude extract from sample #23 to these purified VEA lectins should also be considered. The latter strategy stems from the consideration that molecules capable of limiting biofilm growth and present in the crude extracts of bitter vetch seeds, such as proanthocyanidins (Russi et al. 2019), can provide added value to adhesion limitation of both Gram-positive (Genovese et al. 2021) and Gram-negative human pathogenic bacteria (Ulrey et al. 2014). We believe that the use of plant extracts as multi-component systems, in addition to a lower antibiotic concentration than the one used, may be a reasonable approach for the development of an effective antibiofilm agent.

## Data Availability

The raw data will be available on reasonable request from corresponding author. Sequence data that support the findings of this study have been deposited in the GenBank with the following accession number: PP845299.
